# Multispecies Biofilm Development of Marine Bacteria Implies Complex Relationships Through Competition and Synergy and Modification of Matrix Components

**DOI:** 10.3389/fmicb.2018.01960

**Published:** 2018-08-30

**Authors:** Richard Guillonneau, Claudine Baraquet, Alexis Bazire, Maëlle Molmeret

**Affiliations:** ^1^Laboratoire MAPIEM (EA 4323), Université de Toulon, La Garde, France; ^2^Laboratoire de Biotechnologie et Chimie Marines, EA 3884, l’Institut Universitaire Européen de la Mer, Université de Bretagne-Sud, Lorient, France

**Keywords:** marine bacteria, multispecies biofilm, competition, synergy, matrix components

## Abstract

Microbial communities composition is largely shaped by interspecies competition or cooperation in most environments. Ecosystems are made of various dynamic microhabitats where microbial communities interact with each other establishing metabolically interdependent relationships. Very limited information is available on multispecies biofilms and their microhabitats related to natural environments. The objective of this study is to understand how marine bacteria isolated from biofilms in the Mediterranean Sea interact and compete with each other when cultivated in multispecies biofilms. Four strains (*Persicivirga mediterranea* TC4, *Polaribacter* sp. TC5, *Shewanella* sp. TC10 and TC11) with different phenotypical traits and abilities to form a biofilm have been selected from a previous study. Here, the results show that these strains displayed a different capacity to form a biofilm in static versus dynamic conditions where one strain, TC11, was highly susceptible to the flux. These bacteria appeared to be specialized in the secretion of one or two exopolymers. Only TC5 seemed to secrete inhibitory molecule(s) in its supernatant, with a significant effect on TC10. Most of the strains negatively impacted each other, except TC4 and TC10, which presented a synergetic effect in the two and three species biofilms. Interestingly, these two strains produced a newly secreted compound when grown in dual-species versus mono-species biofilms. TC5, which induced a strong inhibition on two of its partners in dual-species biofilms, outfitted the other bacteria in a four-species biofilm. Therefore, understanding how bacteria respond to interspecific interactions should help comprehending the dynamics of bacterial populations in their ecological niches.

## Introduction

Interspecies interactions appear to have a preponderant role in natural ecosystems. Indeed, the composition of microbial communities, which populate most environments, is largely shaped by interspecies competition or cooperation. Environmental ecosystems are made of various dynamic microscale microhabitats, where microbial communities preferably interact “as metabolically interdependent groups” (Zelezniak et al., 2015; Roder et al., 2016). Bacteria colonize microhabitats compatible with their physiologic and metabolic needs, which depend on their neighboring bacteria and this will influence the spatial structure of the community (Stubbendieck et al., 2016). According to Roder et al. (2016), microscale bacterial interspecies interactions studies are the “missing bridge” between mono and dual species studies and the large-scale investigations focusing on the overall diversity of a community. Microscale interactions studies have the advantage of bringing information on microhabitats and spatial organization as well as bacterial interspecies interactions. Interspecies interactions may also result in different microbial functions and abilities (reviewed in Roder et al., 2016). Very limited information except for the human oral cavities, where multispecies studies are the most abundant (Filoche et al., 2004a,b; Kuboniwa et al., 2006; Kolenbrander et al., 2010; Bloch et al., 2017), are available on microhabitats related to the natural environment (Burmolle et al., 2006; Kuboniwa et al., 2006; Ren et al., 2015; Hansen et al., 2017; Liu et al., 2017). Studying changes in the community composition over the time or what is produced by the population can provide information on the ability of each individual to establish itself and survive in their microhabitat.

As most biofilm communities are composed of multiple different bacteria living in close proximity, embedded in extracellular polymeric substances such as polysaccharides, proteins, nucleic acids, lipids, studying multispecies or multi-organisms biofilms, appears therefore a relevant challenge to take up with the goal to understand how the different populations interact with each others at the microscale level. Numerous obstacles exist in studying mixed biofilms such as labeling the different populations, in particular when they are not amenable to genetic manipulations, or identifying what is produced and by who, interpreting why one strain is better fit than another. Most studies on multi-species biofilms (with four or more species) have recently emerged in the last years but remain overall quite rare (Lee et al., 2014; Ren et al., 2015; Sherry et al., 2016; Bloch et al., 2017; Hansen et al., 2017; Liu et al., 2017). Many studies have used different variants of the same strains or mathematical models and flow systems (Momeni et al., 2013a,b; Lee et al., 2014; van Gestel et al., 2014; Liu et al., 2017), whose flux acting as an additional external factor with a potential influence on the interactions, can be appropriate in certain studies but can restrict quickly space for the organisms on the surface and therefore their spatial organization (Lee et al., 2014; van Gestel et al., 2014; Liu et al., 2017). Multiple species approaches with four or more strains, with a focus on only one or two of the partners, have also been performed, which can bring interesting information on the targeted strains (Bloch et al., 2017; Hansen et al., 2017). Only a few studies following the fate of four (or more) bacterial species in biofilms in interaction, quantitatively (Burmolle et al., 2006; Ren et al., 2015) and on their spatial organization (Lee et al., 2014; Liu et al., 2017) are available. To our knowledge, only one dealt with marine bacterial isolates (Burmolle et al., 2006).

Our study focuses on the comprehension of the interaction within multispecies biofilms of four bacterial strains that have been isolated from immersed biofilms recovered from the Toulon bay (France). The selection of these strains for this study was based on their different phenotypic traits (such as motility, hydrophobicity, adhesion to a surface) and ability to form a biofilm *in vitro* (Camps et al., 2011; Brian-Jaisson et al., 2014; El-Kirat-Chatel et al., 2017; Favre et al., 2017). The objective of this work is to understand how marine bacteria isolated from biofilms in the Mediterranean Sea interact and compete with each other when cultivated together. We investigated whether the outcome of mono or dual species biofilms between these four marine strains cultivated in artificial sea water could give some information on the behaviors they would have in three or four species biofilms. We hypothesized that even if some strains have been described as good or poor biofilm producers in mono-species biofilms, the outcome in a multispecies biofilm, which is not easily predicable, would give valuable information on their ability to colonize and survive in their microhabitats.

## Materials and Methods

### Bacterial Strains and Culture Media

Four marine bacterial strains were used in this study: *Persicivirga (Nonlabens) mediterranea* TC4, *Polaribacter* sp. TC5, *Shewanella* sp. TC10, and *Shewanella* sp. TC11. The strains have been isolated from various surfaces immerged in the bay of Toulon and displayed different phenotypical traits regarding motility, hydrophobicity, adhesion, biofilm formation (Camps et al., 2011; Brian-Jaisson et al., 2014). All bacterial strains were grown in VNSS (Mårdén et al., 1985) at 20°C, 120 rpm. The TC10 strain, which harbored the pX5 plasmid encoding GFP (kindly constructed by Dr. Aurore Puymège), was cultured in VNSS supplemented with 6 μg/mL of chloramphenicol.

### Bacterial Labeling

TC10 pX5-GFP was obtained by conjugation using *E. coli* WM3064 as a donor strain. Briefly, post-exponential phase culture of WM3064 transformed with pX5-GFP was mated with post-exponential phase *Shewanella* sp. TC10 in a 1:1 ratio on VNSS plates containing 100 μg/mL of DAP (2,6-Diaminopimelic acid) (Sigma-Aldrich, St. Louis, MO, United States) overnight at 20°C. *Shewanella* sp. TC10 transconjugants were selected on VNSS DAP free agar plates containing 6 μg/mL of chloramphenicol.

Because the transformation and conjugation assays of the bacterial strains only resulted in the creation of TC10 pX5-GFP despite numerous different conditions tested on the strains, three polyclonal antibodies directed against the three other strains, TC4, TC5, and TC11 were ordered (ACRIS Antibodies GmbH, Herford, Germany) for labeling purposes. Strains were grown to post-exponential phases and washed in PBS 1X by centrifugation at 3700 *g* for 10 min. The pellets were suspended in formalin 0.1% PBS 1× at 4°C during 10 h and washed thrice with PBS 1× prior to obtaining a final concentration of approximately 10^8^ cells/mL before sending them to ACRIS to create their resultant antibodies. The antigens were emulsified with an equal volume of Complete Freund’s Adjuvant and injected into three to four subcutaneous sites for the primary immunization. Subsequent immunizations were performed depending on hosts. TC4, TC5, and TC11 polyclonal antibodies were, respectively, designed into chicken, goat, and rabbit hosts. All the combinations of the primary and the secondary antibodies were tested, and no cross-reactivity was observed (**Supplementary Figure S1**).

### Single Species Biofilm Formation in Static and Dynamic Conditions

For the biofilm formed in static conditions, post-exponential grown bacteria were suspended in ASW (Artificial Sea Water at 36 g/L, Sigma-Aldrich, Darmstadt, Germany) and inoculated at an OD_600 nm_ of 0.1 and 0.3 into 24 well plates (Corning Incorporated Costar^®^, New York, NY, United States) containing a sterilized glass coverslip into each well. At each time point (3, 24, and 48 h), bacteria were washed to remove non-adherent bacteria and fixed using formalin 3.7% during 15 min and permeabilized using Triton^TM^ X-100 (0.05%) during 15 min. The bacteria staining was performed with DAPI at 5 μg/mL (Sigma-Aldrich, Darmstadt, Germany) and the coverslips were mounted with a drop of Prolong^TM^ Diamond Antifade (Thermo Fisher Scientific, Waltham, MA, United States) before observation using epifluorescence microscopy (Zeiss Axiovert 40 CFL, Göttingen, Germany).

Bacterial biofilms were also developed in dynamic conditions on glass slides (Menzel Gläser, Braunschweig, Germany) in ASW in three-channel flow cells (channel dimensions 1 by 4 by 40 mm, Technical University of Denmark Systems Biology, Denmark) as previously performed (Pamp et al., 2009; Bazire et al., 2010). Flow cells were inoculated with bacteria in post-exponential growth phase suspended in ASW at an OD_600 nm_ of 0.3. Adhesion of bacteria occurred at 20°C for 3 h without medium flow, followed by a wash to remove non-attached bacteria using a flow of ASW at 3 mL.h^-1^ during 15 min. The biofilm evolution was monitored with a constant ASW flow of 3 mL.h^-1^ from this stage. At each time point (T3h, T24h, and T48h), the biofilms were stained with 5 μM of Syto 61 red during 20 min and observed by confocal laser scanning microscopy (CLSM, Zeiss LSM 510, and Zeiss LSM 710, Göttingen, Germany).

### Matrix Components Staining

For the matrix staining, a static biofilm of 48 h was performed. Each biofilm was stained with DAPI at 5 μg/mL (Sigma-Aldrich, Darmstadt, Germany) and one of the following matrix dyes. Exopolysaccharides were stained with: (i) a Concanavalin A (ConA) tetramethylrhodamine conjugate (Thermo Fisher Scientific, Waltham, MA, United States) at 20 μg/mL to label Mannose/Glucose residues (Goldstein et al., 1965; Johnsen et al., 2000; Strathmann et al., 2002), (ii) the Wheat Germ Agglutinin (WGA) associated with the Alexa Fluor^TM^ 555 conjugate (Thermo Fisher Scientific, Waltham, MA, United States) at 100 μg/mL to label sialic acids and N-acetyl-glucosamine (Strathmann et al., 2002), and (iii) the Peanut Agglutinin (PNA) lectin from *Arachis hypogaea* peanut associated with the Alexa Fluor^TM^ 568 conjugate (Thermo Fisher Scientific, Waltham, MA, United States) at 50 μg/mL to label mostly glycoproteins which include the Gal-β(1-3)-GalNAc carbohydrate (Angeloni et al., 2005). The proteins were stained with the FilmTracer^TM^ Sypro^TM^ Ruby biofilm matrix stain (Thermo Fisher Scientific, Waltham, MA, United States) according to the manufacturer’s indications and the eDNAs were stained with TOTO^TM^-3 iodide (Thermo Fisher Scientific, Waltham, MA, United States) at 2 μM (Okshevsky and Meyer, 2014). After 30 min of incubation of each probe, each coverslip was washed 3 times in PBS 1×. Finally, the coverslips were mounted with a drop of Prolong^TM^ Diamond Antifade before observation with CLSM.

### Crystal Violet Assay

Bacterial biofilms were developed into 96-well microtiter plates (Greiner Bio-One, Kremsmünster, Austria) with 100 μL of bacteria in post-exponential growth phase in VNSS per well. In parallel, 100 μL of cell-free culture supernatant of another strain were collected in VNSS at the beginning of the stationary growth phase and were added on the bacterial biofilms (the addition of VNSS alone was used as a control). The final OD_600 nm_ was 0.4 into each well. After 48 h of growth in static conditions and a temperature of 20°C, samples were washed thrice with NaCl (36 g.L^-1^) and dried during 30 min at 50°C. Biofilms were stained during 15 min with 200 μL of Crystal Violet at 0.01% (w/v) and rinsed thrice with NaCl (36 g.L^-1^) and dried for 10 min. The quantification of biofilm was evaluated by releasing the stain from the biofilm with absolute ethanol for 10 min at 20°C, at 120 rpm and measuring the absorbance of the Crystal Violet solution at 595 nm. The final OD_595 nm_ of each sample was divided by the blank (i.e., VNSS medium only treated with Cristal Violet).

### Multispecies Biofilm Formation

For the dual-species biofilm, bacteria in post-exponential growth phase were suspended in ASW and inoculated in 24 well plates (Corning Incorporated Costar^®^, New York, NY, United States), containing a sterilized glass coverslip into each well, to a final OD_600 nm_ of 0.3 (0.15 per strain). For the biofilms involving three or four bacterial strains, the final OD_600 nm_ was 0.3 or 0.4 (0.1 per strain). Controls included single species biofilms formed in the same concentrations and conditions than the multispecies biofilm. Biofilm formation was monitored after 24 and 48 h and at each time point, cells were fixed using Formalin 3.7% during 15 min. For the immunostaining, samples were blocked with BSA 3% (Acros Organics, Geel, Belgium) in PBS 1× overnight at 4°C. The primary antibodies were added during 1 h in BSA 3% at 1/300 for Chicken-anti-TC4, 1/100 for Goat-anti-TC5 and 1/300 for Rabbit-anti-TC11. After a second blocking step of 2 h at room temperature, the secondary antibodies were added in BSA 3% to a concentration of 1/2000 for Goat anti-Chicken IgY (H+L) conjugated to FITC (Invitrogen^TM^, Waltham, MA, United States) for 1 h, to a concentration of 1/500 for Goat anti-Chicken IgY (H+L) conjugated to Alexa Fluor 405 (Abcam, Cambridge, United Kingdom) for 1 h, to a concentration of 1/1000 for Donkey anti-Goat IgG (H+L) coupled to Alexa Fluor 633 (Invitrogen^TM^, Waltham, MA, United States) for 1 h, to a concentration of 45 μL/mL for Donkey anti-Rabbit IgG coupled to Alexa Fluor 594 during 30 min and to a concentration of 1/200 for Mouse anti-rabbit IgG coupled to CruzFluor^TM^ 488 for 1 h. Finally, the coverslips were mounted with a drop of ProLong^TM^ Diamond Antifade before observation using CLSM.

### Data Extraction From Images and Statistics

At least three replicates and five pictures per replicate were performed and used for data extraction. The pictures have been acquired by epifluorescence microscopy or by CLSM. The percentages of recovery of epifluorescence microscopy were determined using an algorithmic method with RStudio 0.98.1025 (RStudio, Boston, MA, United States), where the brightness pixel was determined by threshold’s definition of small area of picture around the pixel and against the background. Each picture has been divided in 36 pieces, that permitted empirically to render negligible the distortion of objective and the threshold was defined as two multiplied by percentile 5th of pixel values on a small area in which the pixel was measured (**Supplementary Figure S2**). The biovolume, the average thickness and the evaluation of the maximum coverage in the CLSM pictures was determined with the COMSTAT software developed in MATLAB R2015a (MathWorks, Natick, MA, United States) as previously performed (Heydorn et al., 2000).

To test for statistically significant differences (*P* < 0.05) between two conditions a *t*-test was performed and between different time points, a two-way analysis of variance including the Bonferroni post-test were performed using SPSS 13.0 (IBM, Armonk, NY, United States).

## Results

### Biofilm Formation in Static Conditions

Before studying multispecies biofilm formation, we performed a preliminary experiment in mono-species conditions with the selected four strains in order to evaluate their biofilm formation ability in the conditions selected for the multispecies biofilms. For this purpose, these strains were inoculated onto glass surfaces into the artificial seawater (ASW) medium a nutrient poor solution close to the marine environment composition and their biofilm formation was evaluated in static conditions using an epifluorescence microscope.

**FIGURE 1:**
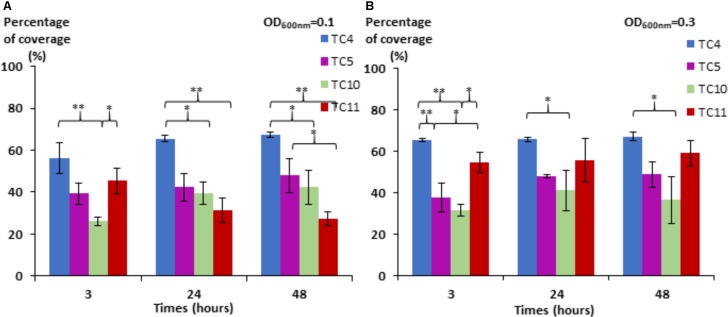
Surface coverage of the four marine bacteria in static conditions. *P. mediterranea* TC4, *Polaribacter* sp. TC5, *Shewanella* sp. TC10, and *Shewanella* sp. TC11 were inoculated in ASW at an OD_600 nm_ of 0.1 **(A)** and 0.3 **(B)** on glass coverslips placed in 24-well plates. After 3, 24, and 48 h of incubation, the bacteria were stained with DAPI, and the epifluorescence pictures were analyzed by an algorithmic method to define the percentage of coverage. Statistical significance was accepted from *p* < 0.05; ^∗^*p* < 0.05; ^∗∗^*p* < 0.01, ^∗∗∗^*p* < 0.001.

We noticed first that in this low nutrient saline solution, all the strains were able to form a biofilm over 48 h with different surface coverages (**Figure 1**). *P. mediterranea* TC4 appeared to have a better surface coverage than *Shewanella* sp. TC10 at both OD_600 nm_ and at all time points and, in a lesser extent, than *Shewanella* sp. TC11 at the OD_600 nm_ of 0.1 and the OD_600 nm_ of 0.3 at 3 h only. The *Polaribacter* sp. TC5 biofilm formation was only significantly different from the one of *P. mediterranea* TC4 at the OD_600 nm_ of 0.3 at 3 h. The concentration of the initial inoculum seemed to have an effect on the TC11 biofilm formation, since it is the only strain with an improved surface coverage at the OD_600 nm_ of 0.3 compared to 0.1. For the other strains, the profiles at each time points appeared similar at both initial OD. Therefore, TC4 displayed the best ability in covering the surface at all time points and initial OD, while TC10 showed the lowest capacity to cover the surface.

### Mono-Species Biofilm Formation Under Dynamic Conditions

**FIGURE 2:**
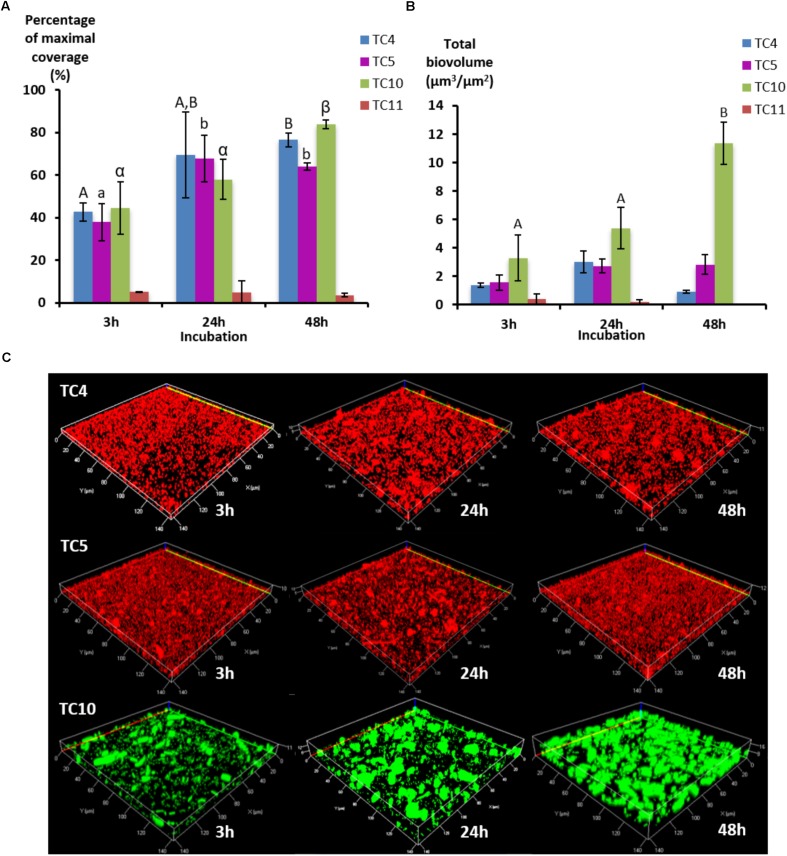
Biofilm formation of the four marine bacterial strains in dynamic conditions. The formation of biofilm was monitored in ASW on flow chamber glass surfaces using a flux of 3 mL/h with *P. mediterranea* TC4, *Polaribacter* sp. TC5, *Shewanella* sp. TC10, and *Shewanella* sp. TC11. After 3, 24, and 48 h of incubation, the bacteria were stained with Syto 61 or directly imaged for TC10 pX5-GFP. **(A)** The percentages of coverage of the most occupied slice were defined over time by the COMSTAT algorithm. **(B)** The biovolumes were calculated at the same time points by the COMSTAT algorithm. **(C)** Representative pictures of the biofilm formed after 3, 24, and 48 h of incubation are shown. Values are the mean of three replicates, errors bars represent standard deviation and significant differences between incubation times (*p*-value <0.05) are indicated by different letters **(A,B)** in a same alphabet (A≠a≠α).

In order to study if these strains would behave, similarly, in dynamic conditions, the same experiment was performed using a flow system set at 3 mL/h and images were analyzed at 3, 24, and 48 h. To obtain a sufficient coverage of the surface in dynamic conditions, the OD_600 nm_ of 0.3 was chosen. The results show that the biofilm of the four marine strains presented different patterns over the time compared to the ones observed when in static conditions, which could be at least for *Shewanella* sp. TC11 attributed to the flux (**Figure 2A**). Indeed, *Shewanella* sp. TC11 detached itself very rapidly from the surface with the instauration of the flux, as TC11 had less than 5% of surface coverage at 3 h. The other strains managed to adhere more efficiently with the flux, covering the surface at around 40% at 3 h. While in static conditions, TC4 displayed a surface coverage above 60% at 3 h (**Figure 1**), it was only around 40% in dynamic conditions (**Figure 2A**). The difference between the surface coverages in static versus dynamic conditions at 3 h was less important for *Polaribacter* sp. TC5 and *Shewanella* sp. TC10. This suggests that TC4 was also impacted by the flux at least at the first time point. The surface coverage of TC4, TC5, and TC10 increased slowly over the 48 h in contrast with the mono-species biofilm. The biovolumes were significantly higher for TC10, in particular at 48 h, compared to the other strains (**Figure 2B**). This could be attributed to the observation of bigger patches of filamentous bacteria, in sharp contrast with the thinner regular homogeneous repartition of TC4 and TC5 on the surface (**Figure 2C** and data not shown). Indeed, TC5 and TC4 appeared to form a biofilm that covered most of the surface (at least similar to TC10) but without the presence of 3D structures (**Figure 2C**).

### Bacteria Appear to be Specialized in the Secretion of Few Exopolymeric Compounds

Because TC11 was not able to form a real biofilm in dynamic conditions, the following experiments were performed in static conditions at an OD_600 nm_ of about 0.1 per strain in order to avoid a too high overall inoculum and the influence of the flux on bacterial attachment, which may hide the inhibition of biofilm formation in multispecies conditions.

Before performing multispecies biofilms, and in order to better understand the different characteristics of the biofilm formation of these strains, fluorescent markers were used to label major compounds of the extracellular matrix (Goldstein et al., 1965; Neu and Lawrence, 1999; Johnsen et al., 2000; Strathmann et al., 2002; Angeloni et al., 2005; Kuboniwa et al., 2006; Okshevsky and Meyer, 2014).

**FIGURE 3:**
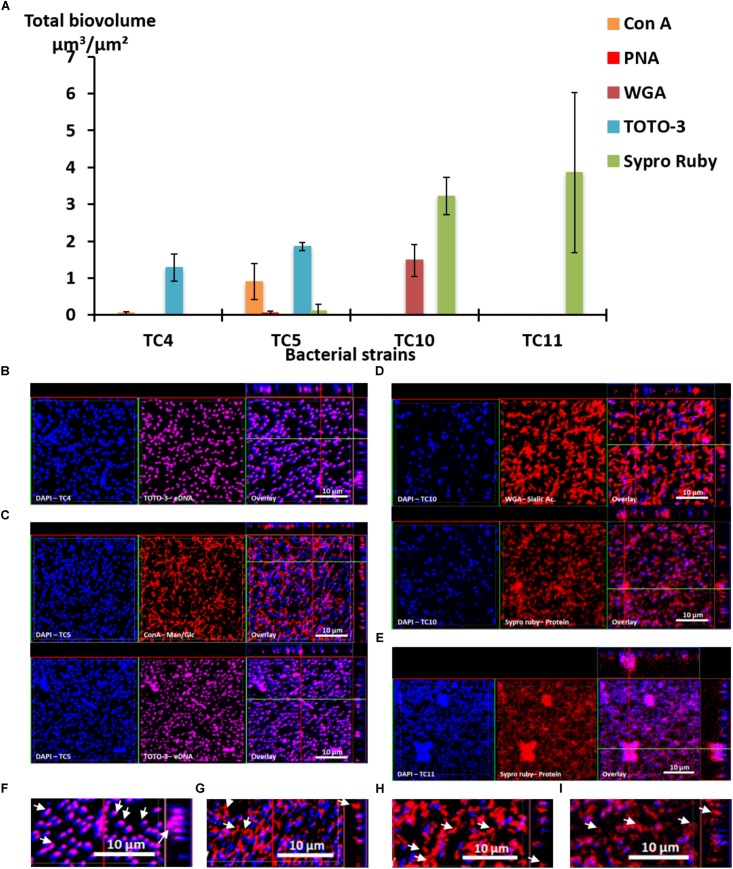
Matrix exopolymers secreted by the marine bacterial strains in static and single species conditions. Exopolymeric substances secreted in the biofilm for each of the four strains were assessed in ASW after 48 h. Different fluorescent markers were used: Concanavalin A for exopolysaccharides of glucose and/or mannose, WGA lectins for exopolysaccharides of sialic acids, PNA lectins for exopolysaccharides containing glycoproteins, TOTO3 for eDNA and Sypro Ruby for proteins. **(A)** The biovolumes of each matrix compound were evaluated by the COMSTAT algorithmic method. Representative pictures of the observed secretion of matrix compounds are presented for *P. mediterranea* TC4 **(B)**, *Polaribacter* sp. TC5 **(C)**, *Shewanella* sp. TC10 **(D)**, and *Shewanella* sp. TC11 **(E)**. A closer view of the TC4 biofilm labeled with TOTO3 **(F)**, the TC5 biofilm labeled with ConA **(G)**, the TC10 biofilms labeled with WGA **(H)**, and Sypro Ruby **(I)** are presented. The white arrows indicate the localization of the matrix compounds. Values are the mean of three replicates, errors bars represent standard deviation.

First, the results show that each strain presented a specific pattern of production of the studied matrix compounds. *P. mediterranea* TC4 produced mainly eDNA (labeled with TOTO-3) in its matrix. As shown in the **Supplementary Figure S3**, the production of components made of glucose or mannose residues corresponded more to a background noise similar to the detection of glycoproteins by PNA than a real production of exopolymers. *Polaribacter* sp. TC5 produced eDNA and exopolysaccharides composed of glucose or mannose residues as shown by the ConA labeling (**Figure 3A**). For these two strains, eDNA was the major secreted compound. The two *Shewanella* strains TC10 and TC11 produced mainly proteins in their matrix visualized with the Sypro Ruby staining, but TC10 secreted also sialic acid residues or N-acetyl-glucosamine as shown by the WGA labeling. Overall, among the extracellular matrix compounds tested, most of the compounds secreted by these bacteria were eDNA or proteins. Interestingly, when observing CLSM images, TC4 appeared to secrete eDNA at the upper biofilm part as seen on the Z section images, similar, to TC5 (**Figures 3B,C,F**). TC5 produced also polysaccharides that are composed of glucose and mannose, which appeared as thin filaments and are distributed on the surface without being necessarily directly associated with the bacterial body (**Figures 3C,G**). This suggests that these polysaccharides can be entirely secreted outside of the cell, without remaining attached to it. Similarly, for TC10, exopolysaccharides were composed of sialic acids residues or N-acetyl-glucosamine, which appeared as big patches and some of these patches seemed to be separated from the TC10 bacterial body (**Figures 3D,H**). The exopolymeric substances composed of proteins for TC10 and TC11 appeared overall smaller compared to the secreted exopolymers composed of sialic acids. They are also less homogeneous in size with the presence of patches (**Figures 3D,E**). In the bacteria tested, none of them secreted compounds tagged by PNA.

Taken together, these results show that each bacterial strain is able to secrete different compounds in their matrix. Among the extracellular matrix compounds tested, some bacteria secreted only one of the compounds, such as TC4 with eDNA or TC11 with proteins. Thus, these bacteria were specialized in the secretion of one or two types of exopolymeric compounds in their matrix.

### Most of the Bacteria Exert an Inhibitory Effect on Their Partners When They Are in Presence of Each Other

In order to study how these strains behave when they are in presence of each other, we first examined if some of the bacterial strains secreted antibiofilm molecules by testing their supernatants against each other’s biofilms using the Crystal Violet assay. As all the strains can form a biofilm in ASW but cannot grow planktonically in this medium, these experiments were performed using the VNSS marine medium.

**FIGURE 4:**
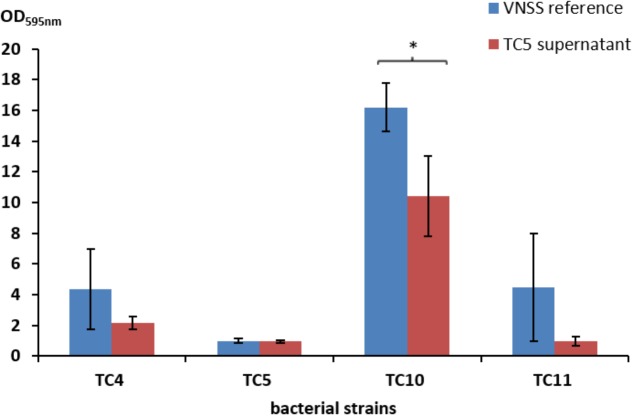
Antibiofilm activity of the TC5 supernatant on the marine bacteria biofilms. The biofilm formation of *P. mediterranea* TC4, *Polaribacter* sp. TC5, *Shewanella* sp. TC10, and *Shewanella* sp. TC11 was evaluated by the Crystal Violet staining approach in VNSS with or without (VNSS Reference) the addition of the TC5 supernatant harvested in stationary growth phase after 48 h in static conditions. Values are the mean of three replicates, error bars represent standard deviations and asterisks indicate significant difference to the VNSS reference (^∗^*p* < 0.05 and ^∗∗^*P* < 0.01).

Most strain supernatants had no efficiency in limiting biofilm formation of the other strains, except for the supernatant of *Polaribacter* sp. TC5 (**Figure 4** and **Supplementary Table S1** and data not shown). When harvested in the early stationary phase, the TC5 supernatant limited biofilm formation of *Shewanella* sp. TC10. None of the supernatant induced a pH modification of the medium (data not shown). These results showed an anti-biofilm effect of the TC5 supernatant on one of the tested strains.

In order to better understand the antagonist or synergetic interactions existing between the bacteria within their biofilms and to verify if TC5 could exert a similar anti biofilm effect against one of its potential competitors in an actual biofilm, dual-species biofilms were performed and studied after 24 and 48 h using CLSM.

**FIGURE 5:**
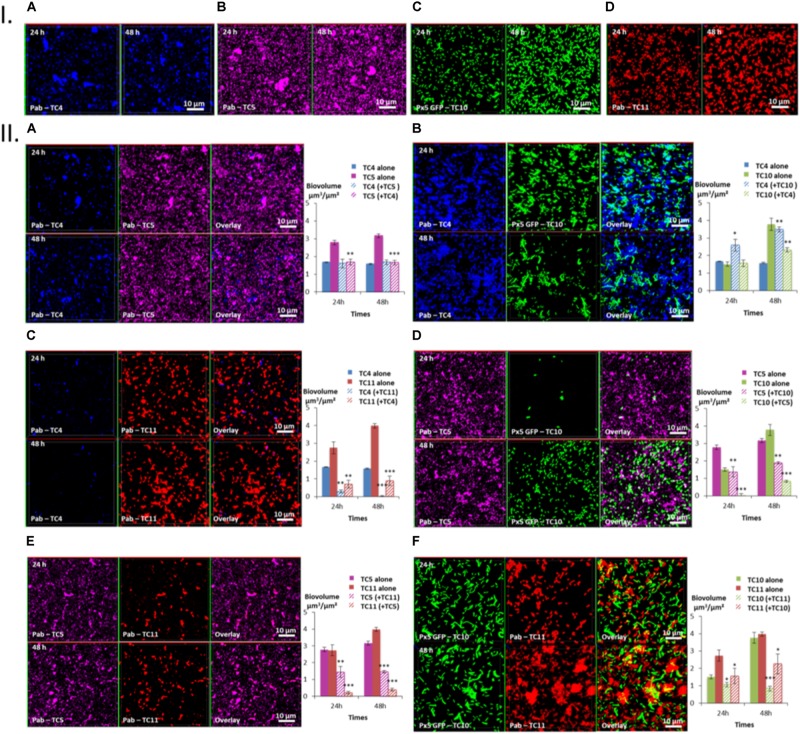
Biovolumes and spatial patternings of bacteria in dual-species biofilms. The two species biofilm formations were analyzed through each combination of the four bacterial strains in ASW after 24 or 48 h of incubation. **(IA–D)** Images of the single-species biofilms with *P. mediterranea* TC4, *Polaribacter* sp. TC5, *Shewanella* sp. TC10, and *Shewanella* sp. TC11 in ASW. **(IIA–F)** Spatial patterning and biovolumes of each strain in dual-species biofilms compared to when cultivated alone. Values are the mean of three replicates, error bars represent standard deviations and asterisks indicate significant difference to the strain incubated alone (^∗^*p* < 0.05, ^∗∗^ *P* < 0.01, and ^∗∗∗^*P* < 0.001).

Three patterns of interactions could be identified: The first type of interaction is illustrated by the TC4/TC5 dual-species biofilm (**Figure 5IIA**). An inhibitory effect was observed on the TC5 biofilm while no effect was noticed for TC4 compared to their respective mono-species biofilm at both time points (**Figures 5IA,B**). Their biofilm biovolume did not evolve between 24 and 48 h. The second type of interaction is associated with the TC4/TC10 dual-species biofilm (**Figure 5IIB**), where the TC4 biofilm appeared stimulated compared to the mono-species conditions (**Figures 5IA,C**) while the TC10 biofilm was affected at 48 h. However, both biofilms grew between 24 and 48 h. The third type of interaction involved all the other dual-species biofilms (**Figures 5IIC–F**), which showed a diminution of the biofilm biovolume for both strains at 24 and 48 h compared to their mono-species biofilms. Despite the inhibition of their biofilm compared to their mono-species biofilm biovolumes, their biofilm evolved differently between 24 and 48 h. For the TC4 and TC11 dual-species biofilm, the TC4 biofilm decreased over the time while the TC11 biofilm stagnated. In the case of TC5 and TC10, both biofilms were able to regrow between 24 and 48 h. In the TC5 and TC11 biofilm, the biovolume stayed at the same level over time. At last for the two strains of *Shewanella*, the TC10 biofilm was even more impacted at 48 h while this of TC11 was able to regrow over time.

Taken together, these results show that *P. mediterranea* TC4 is the only bacterial strain which appeared to benefit from a partner, here *Shewanella* sp. TC10, and to not be impacted by the presence of TC5. Overall, TC4 seems to cope better with the presence of the other bacteria since only TC11 has a negative impact on this strain. In contrast, TC4 has a negative impact on TC5, TC10, and TC11. *Polaribacter* sp. TC5 is affected by the presence of all the other strains and appears to inhibit the biofilms of TC10 and TC11 but not the one of TC4. *Shewanella* sp. TC10 is impacted by all the strains. While it reduces the biofilm biovolumes of TC11 and TC5, it appears to help TC4 forming more biofilm. It is interesting to note that the shape of TC10 varies according to the situation, appearing bigger and/or filamentous with TC4 and TC11 than when cultivated with TC5 or alone. At last, *Shewanella* sp. TC11 is impacted by all the other partners but appears to induce a negative effect on them too. It is interesting to note that TC5 induces two of the biggest diminution of biofilm biovolumes on TC10 (Δ-2.94 μm^3^/μm^2^) and on TC11 (Δ-3.6 μm^3^/μm^2^), while TC4 induces only one important diminution on the TC11 biofilm (Δ-3.1 μm^3^/μm^2^) and TC11 induces another one on the TC10 biofilm (Δ-2.94 μm^3^/μm^2^). Therefore, even though, TC5 negatively impacts only two strains TC10 and TC11, both effects are among the strongest. The fact that only the supernatant of TC5 seems to have an inhibitory effect on TC10 suggest that this inhibition may be due to the secretion of molecule(s) in the supernatant. Whether TC5 is able to secrete these component(s) in ASW and in presence of other bacteria is not known.

### The Dual-Species Biofilm of TC4 and TC10 Unravels the Production of a New Secreted Compounds in the Matrix Compared to When Grown Independently

**FIGURE 6:**
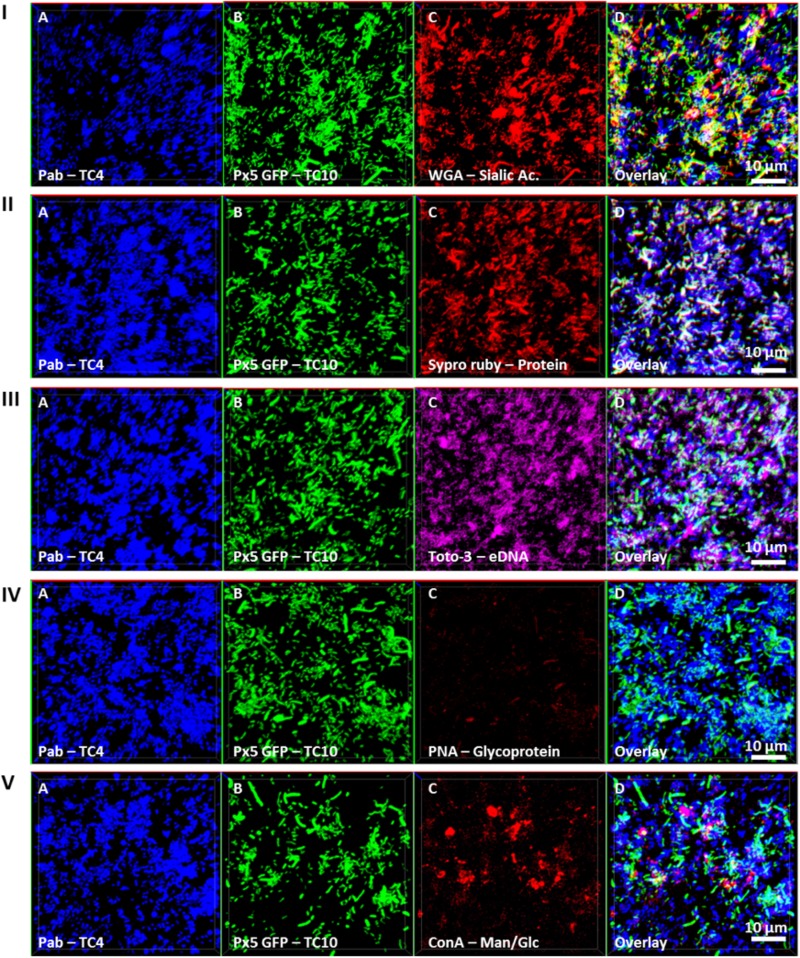
Matrix Exopolymers secreted in the dual-species biofilm of TC4 and TC10. The exopolymeric substances secretion in the TC4 and TC10 biofilm was assessed in ASW after 48 h of incubation. The exopolymeric components were tagged using the following fluorochromes: exopolysaccharides of sialic acids with WGA lectins **(I)**, proteins with Sypro Ruby **(II)**, eDNA with TOTO3 **(III)**, exopolysaccharides containing glycoproteins using PNA lectins **(IV)**, and exopolysaccharides of glucose and/or mannose using Concanavalin A **(V)**.

In order to investigate if the increase of the biofilm formation of *P. mediterranea* TC4 (and in a lesser extent of *Shewanella* sp. TC10) could be due to a modification of the matrix composition, different compounds of the matrix were tagged with fluorescent markers after 48 h of biofilm formation and analyzed using CLSM (**Figure 6**). The results showed that when the two strains were together, sialic acids residues or N-acetyl-glucosamine (labeled by WGA) were detected (**Figure 6I**) as well as proteins (labeled by Sypro Ruby) similar to when TC10 was alone (**Figures 3**, **6II**). The production of eDNA was also observed similar to when TC4 was alone (**Figure 6III**). No production of glycoprotein or β-galactose residues were detected by PNA as it was also the case when both strains were grown alone (**Figure 6IV**). Surprisingly, the association of the two bacteria triggered the production of exopolysaccharides made of mannose and glucose (detected by ConA), while these exopolysaccharides were not observed in the mono-species biofilms (**Figure 6V** and **Supplementary Figure S2**). Therefore, it is possible that the association between TC10 and TC4 leads to the production of compounds made of glucose and/or mannose residues in the matrix that may be responsible for the TC4 increased biofilm.

### The Inhibitory Effect of TC11 Does Not Affect the Beneficial Interactions Between TC10 and TC4

In order to know if the synergetic effect detected in the *P. mediterranea* TC4 and *Shewanella* sp. TC10 dual-species biofilm could take place in a three-species biofilm and could be inhibited by *Shewanella* sp. TC11, TC11 was added to the TC4 and TC10 dual-species biofilm, as it is the only strain able to impact both TC4 and TC10. Similar to the dual-species biofilms, TC4, TC10, and TC11 were inoculated in the same proportion on to glass coverslips and the biofilms were studied at 24 and 48 h using CLSM.

**FIGURE 7:**
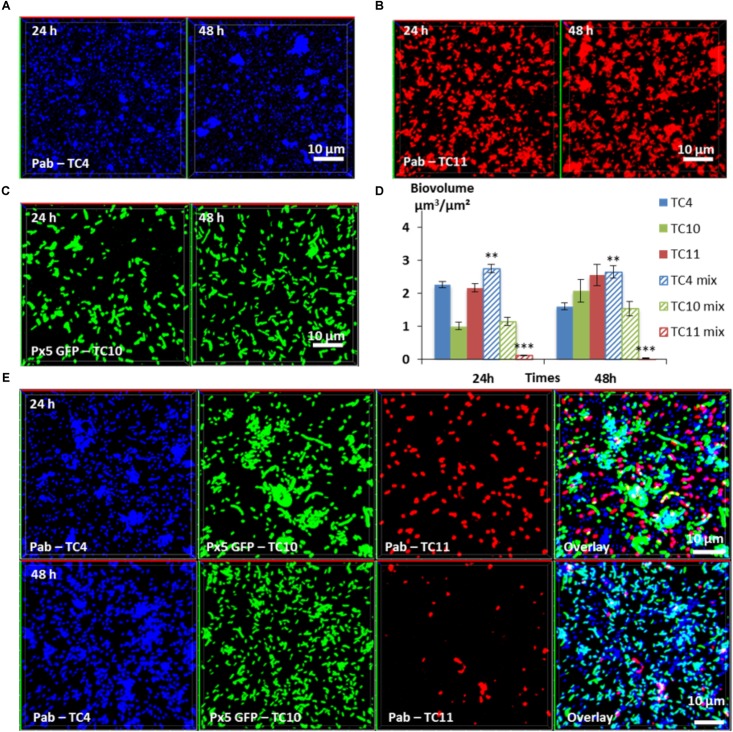
Biovolumes and spatial patternings of bacteria in three-species biofilms. The three-species biofilm formation selected including *P. mediterranea* TC4, *Shewanella* sp. TC10, and *Shewanella* sp. TC11 was analyzed in ASW after 24 or 48 h of incubation. Representative **i**mages of the single-species biofilms with *P. mediterranea* TC4 **(A)**, *Shewanella* sp. TC10 **(B)**, and *Shewanella* sp. TC11 **(C)**. **(D)** Biovolumes of each strain in three species biofilms compared to when cultivated alone. **(E)** Representative spatial patterning images after 24 or 48 h of bacteria in three species biofilms. Values are the mean of three replicates, error bars represent standard deviations and asterisks indicate significant difference to the strain incubated alone (^∗∗^*p* < 0.01 and ^∗∗∗^ *P* < 0.001).

In the three-species biofilm comprising TC4, TC10, and TC11, TC4 still presented a significant increase of the biofilm biovolume at 24 and at 48 h (**Figure 7D**). Thus, the presence of TC11, which was the only strain able to negatively impact the TC4 biofilm in the dual-species biofilm, did not induce a diminution of the TC4 biovolumes in presence of the three strains. While the TC4 ability to adhere on the surface strongly decreased at 24 h in the TC4 and TC11 dual-species biofilms (**Figure II5C**), here the biovolume of TC4 at 24 h was higher than in the TC4 mono-species biofilm. This could suggest a synergetic effect of TC10 stimulating and protecting TC4 against TC11, may be through the production of exopolymers containing glucose and/or mannose residues. In the three-species biofilm, the biovolume of TC10 increased slightly between 24 and 48 h, while the one of TC11 diminished significantly. The morphology of TC10 at 24 h, which presented the same distinctive big and/or filamentous shapes than in the dual-species biofilm with TC11, changed toward smaller bacteria at 48 h, similarly, to when cultivated alone (**Figures 7E**, **5IC**). This is probably due to the reduction of the presence of TC11, similar to the results observed in the dual-species biofilms, where TC11 seemed to trigger filamentation of TC10 (**Figures 5IID,F**). Since TC11 was impacted by all the bacteria in the dual-species biofilms, the TC11 biofilm decreased sharply in the three-species biofilm, probably under the effect of both TC10 and more importantly TC4 (**Figure 7D**). Therefore, in this situation, it is TC11 that suffered the most from the presence of the two other partners, even though TC11 was able to negatively impact TC10 and in a lesser extent TC4 in the dual-species biofilms.

### TC5 Outfits Its Competitors in a Four-Species Biofilm

**FIGURE 8:**
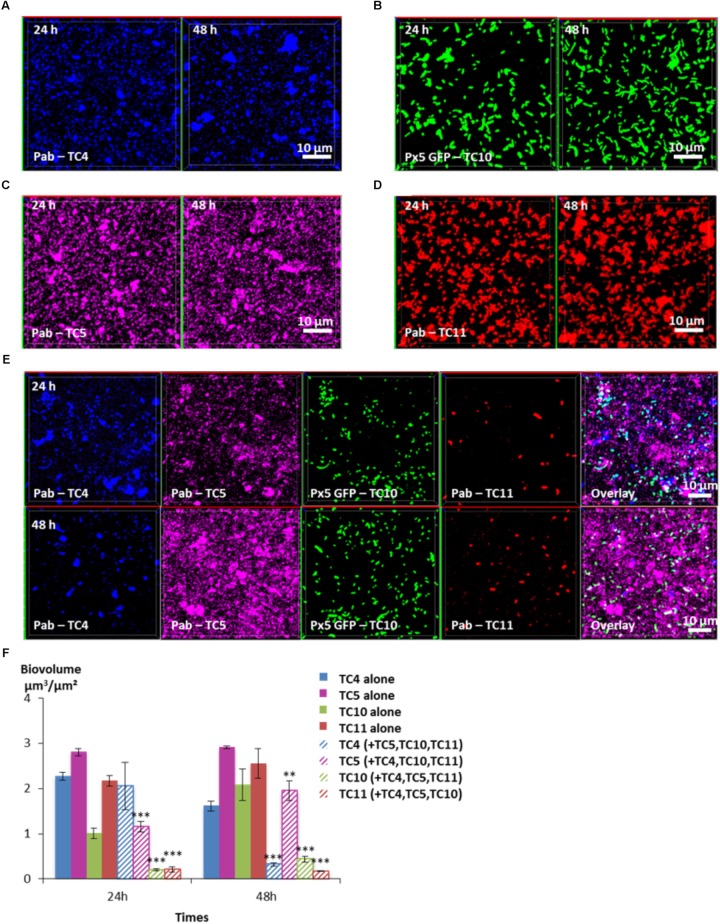
Biovolumes and spatial patternings of bacteria in four-species biofilms. The four species biofilm formation including *P. mediterranea* TC4, *Shewanella* sp. TC10, *Shewanella* sp. TC11, and *Polaribacter* sp. TC5 was analyzed in ASW after 24 or 48 h of incubation. Representative mages of the single-species biofilms with *P. mediterranea* TC4 **(A)**, *Polaribacter* sp. TC5 **(B)**, *Shewanella* sp. TC10 **(C)**, and *Shewanella* sp. TC11 **(D)**. **(E)** Representative spatial patternings after 24 or 48 h of development in four-species biofilms. **(F)** Biovolumes of each strain in four species biofilms compared to when incubated alone. Values are the mean of three replicates, error bars represent standard deviations and asterisks indicate significant difference to the strain incubated alone (^∗∗^*p* < 0.01 and ^∗∗∗^*p* < 0.001).

In order to know which of the four bacterial strains would overtake the other ones in a four-species biofilm, the biofilm comprising the four bacteria inoculated in the same proportion onto glass coverslips was monitored using CLSM at 24 and 48 h (**Figure 8**). After 24 h, the TC4 biovolume was not modified in the presence of its three other partners compared to its mono-specific biofilm (**Figures 8A,E,F**), similarly, to the results obtained in the dual-species and the triple-species biofilms. For all the other strains, the biofilm biovolumes decreased sharply at 24 h. However, at 48 h, TC4 decreased strongly while TC11 maintained its low-biovolume biofilm at the same level between 24 and 48 h in the four-species biofilm. While TC10 was subjected to a small but significant increase, it is TC5 which displayed the more important growth at 48 h compared to 24 h. Therefore, even though, they were all impacted compared to their respective mono-species biofilms, TC5 was able to regain a stronger biofilm at 48 h. Since TC5 displayed a strong inhibition on TC11 and TC10 in the dual-special biofilms, while having no effect on TC4, whose growth is, however, impacted by the diminution of TC10 biofilm, it might not be surprising that TC5 ended up being the fittest bacteria in this four-species biofilm.

## Discussion

In this study, we decided to pursue the comparison between the different biofilm formation abilities of 4 of the marine strains previously isolated from the Mediterranean sea (Camps et al., 2011; Brian-Jaisson et al., 2014), with the objectives to understand to which extent their capacity of adhesion and biofilm formation are different in other conditions (low-nutrient medium, glass surfaces…) and how they could interact and compete with each other when cultivated in multispecies biofilms. In order to get closer from the natural marine environment and to induce metabolic interactions between the bacterial strains, artificial sea water was used for all the experiments. To set up multispecies biofilms, we selected four strains with different phenotypical particularities and abilities to adhere and to form biofilms (Brian-Jaisson et al., 2014; El-Kirat-Chatel et al., 2017), hypothesizing that even if in some conditions bacteria appear to produce more biofilms than others, the outcome in a multispecies biofilm is not easily predictable, due to the existence of multiple types of interactions (antagonistic, synergetic…). TC4 a newly identified species of *P. mediterranea*, and TC5 a *Polaribacter* strain, characterized by a higher hydrophobicity, both non-motile rods, produced small biofilms when cultivated in microplates and rich media. Two motile bacilli belonging to the *Shewanella* genus, TC10 and TC11 appeared to produce more biofilm in the same conditions (Brian-Jaisson et al., 2014). In addition, TC11 formed an important biofilm when inoculated in ASW on glass surface at high OD_600 nm_, compared to TC5 and TC10 (El-Kirat-Chatel et al., 2017). However, using Atomic Force Microscopy (AFM), TC11 appeared to display a phenotypic heterogeneity in the very early stage of the adhesion in sharp contrast with TC5 or TC10, whose individuals within the bacterial population presented more homogeneous adhesion forces (El-Kirat-Chatel et al., 2017).

In this study, we show that the strain, which adheres and covers the most the surface when in mono-species and static conditions is a strain initially described as a poor biofilm producer, *P. mediterranea* TC4 (Brian-Jaisson et al., 2014). Overall, the development of bacterial biofilms of most strains within the multi-species biofilms appears to be inhibited compared to the development of their respective single-species biofilm, with the exception of TC4. In the two- and three-species biofilms, this strain is the only one able to benefit from its interaction with one of its partners, here *Shewanella* sp. TC10 (**Figures 5IIB**, **7**). In the four-species biofilm, *Polaribacter* sp. TC5 outfits all the other strains, including TC4, but its biofilm biovolume is still below the one measured in its mono-species biofilm (**Figure 8**). In contrast, the strain which appears to be overall the most impacted is *Shewanella* sp. TC11. Indeed, in the dual-species biofilms, TC11 appears to be impacted negatively by all the strains and to be outfitted by its partners in the three- and four-species biofilms. However, since none of the TC11 cells have completely disappeared at 48 h, it is possible that due to its potential phenotypical heterogeneity (El-Kirat-Chatel et al., 2017), presumably allowing an increased bacterial fitness, the cells of this strain could become a tolerant slow-growth subpopulation and/or could play a role in the overall growth fitness and spatial organization of the multispecies biofilm as it has been recently shown (Grote et al., 2015; Liu et al., 2017). Specific sub-population of persister bacteria corresponding to phenotypic variants, which emerge under stressful conditions such as nutrient limitation, antibiotic pressure, or transition from planktonic to biofilm state, may be still present in small proportions in the biofilm. On another hand, TC11 might simply be less resistant to harsh or competitive conditions such as in multispecies biofilms than the other strains. In our study, we only observed the initial stage of the biofilm development in relation to the initial interactions. A longer incubation time in ASW might have given more insights on bacterial competition and spatial organization. However, mechanisms explaining how interspecies interactions affect spatial organization are poorly understood as this may correspond to complex and multiples dynamics sequential events involving many external parameters. Growth of some strains might be initially affected by both cooperative and competitive interactions, but this interrelationship might change over time with the development of the community in response to both cell–cell interactions and environmental factors (Liu et al., 2016).

In our case, the only strain which appears to secrete molecule(s) with an anti-biofilm activity is *Polaribacter* sp. TC5 (**Figure 4**). We show that the TC5 supernatant inhibits the *Shewanella* sp. TC10 biofilm but not the one of *Shewanella* sp. TC11, although TC5 is able to inhibit both *Shewanella* in their dual-species biofilms (**Figures 5IID,E**). This suggests that in dual-species biofilms, secreted molecule(s) in the extracellular medium of TC5 could be involved in the TC10 inhibition, while a direct cell–cell interaction might also be required for the TC11 inhibition. Therefore, in our experiments, TC5 is potentially able to inhibit the two *Shewanella* strains TC10 and TC11 biofilms through different mechanisms. Whether this secretion has helped TC5 to outcompete the others strains in the four-species biofilm in particular at 48 h, is not known (**Figure 8**). Some bacteria may secrete enzymes that could interfere with mechanisms involved in this secretion or counteract the secreted effectors and therefore could interfere with a specific population beneficial phenotype (reviewed in Sanchez-Vizuete et al., 2015; Wen et al., 2014). In addition, direct or physical interactions between bacteria, which involve for instance horizontal gene transfer, known to improve adaption and to originate new ecological populations (reviewed in Wiedenbeck and Cohan, 2011), could have taken place between the isolates, inducing different phenotypical consequences on the receiver(s) (Sanchez-Vizuete et al., 2015). Although, we have tried repeatedly to transform the four strains with plasmids carrying GFP without success, except for TC10, this does not mean that gene transfer cannot occur in these biofilms conditions. It is interesting to note that TC4 and TC5 secrete mainly eDNA in their matrix in sharp contrast with TC10 and TC11 and that in the TC10 and TC4 dual-species biofilms, eDNA was still present. Overall, how these mechanisms take place within multispecies biofilms are difficult to decipher and poorly studied up to now.

In our study, TC4 and TC10, produce a newly secreted compound (exopolysaccharide made of mannose and glucose residues), that is not present in their respective mono-species biofilm. This compound may be responsible for the TC4 increased biofilm, as it has been previously shown with other bacteria (Zogaj et al., 2001). Even when TC11 is added to the dual-species biofilm of TC4 and TC10, the inhibitory effect of TC11 observed independently in the dual-species biofilms toward TC10 and TC4, does not affect the beneficial interactions between TC10 and TC4 in the three-species biofilm. Cooperative phenotypes could include the production of exopolymers in the biofilm matrix, which can enhance biofilm-related properties, such as resistance or growth. Secreted molecules can also be considered as cooperative public goods as they can be utilized by bacteria that are not producing them. The mechanisms by which public goods-producing bacteria avoid exploitation in biofilm is not fully understood. Public goods usually benefit nearby clonemates and factors such as restricted diffusion of nutrients, inoculation under flow or at low density are benefiting the public goods producing bacteria (Drescher et al., 2014; van Gestel et al., 2014). While studies on the mechanisms involved in competition or synergy are rare in multispecies biofilms, a transcriptomic study focusing on gene expression analyses in multispecies biofilms involving *Xanthomonas retroflexus*, has recently shown that the majority of the genes only regulated in the four strains biofilm were downregulated. Interestingly, this suggests that *X. retroflexus* seems to deprioritize vital functions for mono species cultures, as partners may share products from energy-consuming pathways in the four-species biofilm (Hansen et al., 2017). Therefore, metatranscriptomic approaches are interesting tools that can help deciphering some of the interaction mechanisms involved multispecies biofilms.

## Conclusion

This study highlights three main observations regarding bacterial interactions in multispecies biofilms. First, most of the marine strains inhibited each other in the two-, three-, and four- species biofilms. Second, one of the strain was able to benefit from one of its partners in the two- and three- species biofilms. In dual-species biofilms, they were able to produce an additional matrix compound. Third, the bacterial strain that was able to induce the strongest inhibition on several of its partners in dual-species biofilms, while being inhibited in a four-species biofilm, outfitted the other strains. These results confirm the importance of studying competitive bacterial interactions in multispecies biofilms and conditions close to the ones encountered in their natural environment to unravel the complexity of dynamics of bacterial population in their microhabitats.

## Author Contributions

RG performed all the experiments of this article under the direction of MM and the co-direction of CB. The flow experiments were performed under the direction of AB in the LBCM Laboratory, Université de Bretagne-Sud, in Lorient. With the guidance of AB, RG set up the flow system experiments in the MAPIEM Laboratory, Université de Toulon, La Garde, extracted and analyzed data from the related CLSM images.

## Conflict of Interest Statement

The authors declare that the research was conducted in the absence of any commercial or financial relationships that could be construed as a potential conflict of interest.
