# Supplementary material for: Multispecies Biofilm Development of Marine Bacteria Implies Complex Relationships Through Competition and Synergy and Modification of Matrix Components
Source: Front Microbiol. 2018 Aug 30;9:1960. doi: 10.3389/fmicb.2018.01960 (PMC6125326; doi:10.3389/fmicb.2018.01960)
Supplement: Supplementary file 1 [file Table_1.docx]

Supplementary Material

**Multispecies biofilm development of marine bacteria implies complex relationships through competition and synergy and modification of matrix components**

**Guillonneau Richard^1^, Baraquet Claudine^1^, Bazire Alexis^2^ and Molmeret Maëlle^1^**

**Correspondence :**

Maëlle Molmeret, Laboratoire MAPIEM EA 4323, Université de Toulon

E-mail: molmeret@univ-tln.fr

# Supplementary Table S1

**Table S1. Effect of the supernatant of each strain on each other’s biofilms**

|  | **Effect on biofilm formation** | | | |
| --- | --- | --- | --- | --- |
| **Supernatant** | **TC4** | **TC5** | **TC10** | **TC11** |
| TC4 | 0• | -0.1 | +0,1 | +0.1 |
| TC5 | -2.2 | -0.1 | -5.8 * | -3.5 |
| TC10 | -0.7 | +2.0 | +2.6 | +0.7 |
| TC11 | -0.5 | -1.1 | +1.0 | -0.5 |

• Values are as follow: [(OD_595nm_Strains/OD_595nm_Blank)_Biofilm with VNSS reference_] - [(OD_595nm_Strains/OD_595nm_Blank)_Biofilm with strain supernatant_]

* Statistically significant value accepted at *P*<0.05
