# Supplementary material for: Multispecies Biofilm Development of Marine Bacteria Implies Complex Relationships Through Competition and Synergy and Modification of Matrix Components
Source: Front Microbiol. 2018 Aug 30;9:1960. doi: 10.3389/fmicb.2018.01960 (PMC6125326; doi:10.3389/fmicb.2018.01960)
Supplement: Supplementary file 2 [file Presentation_1.pptx]

## Slide 1
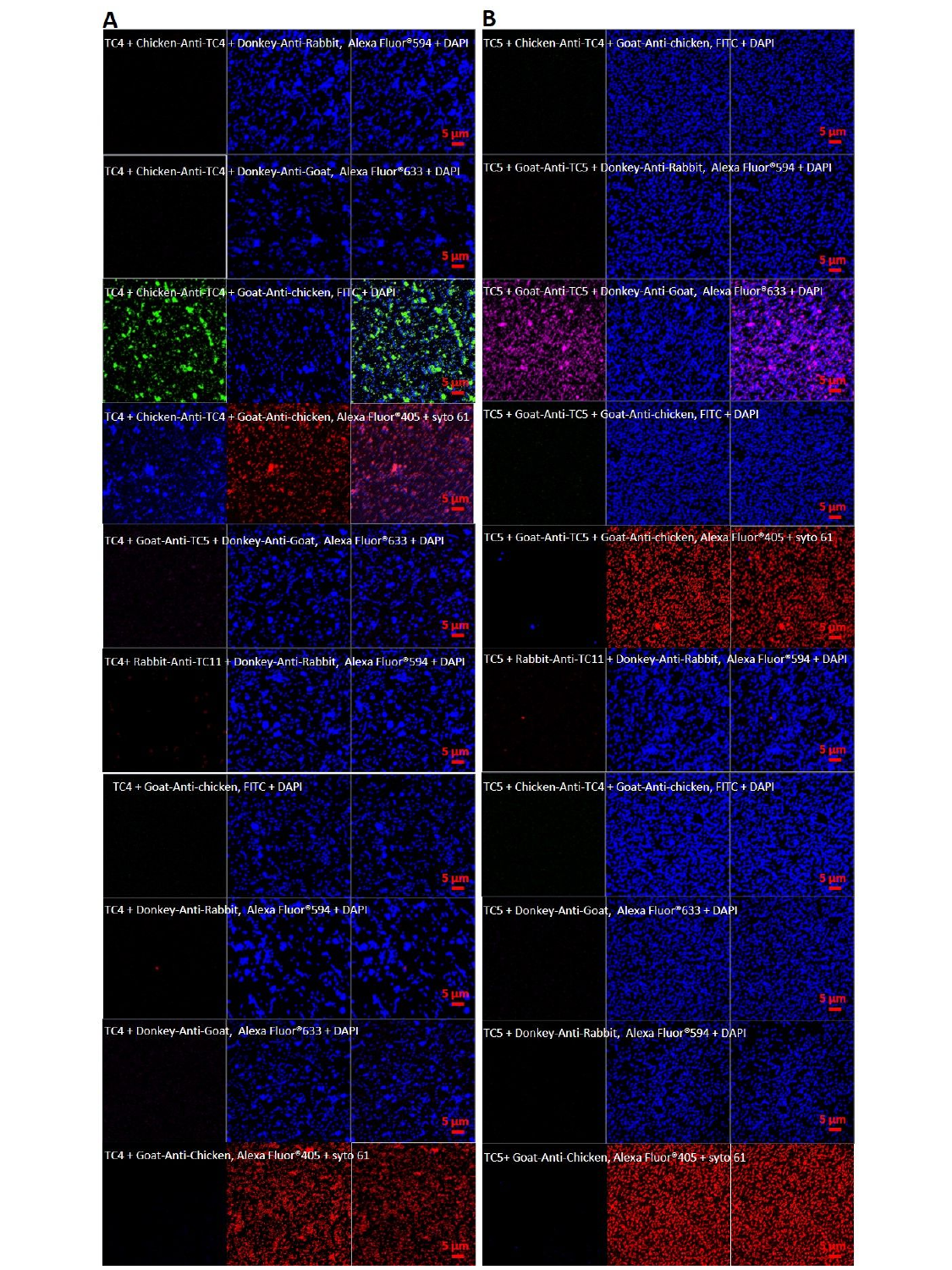

## Slide 2
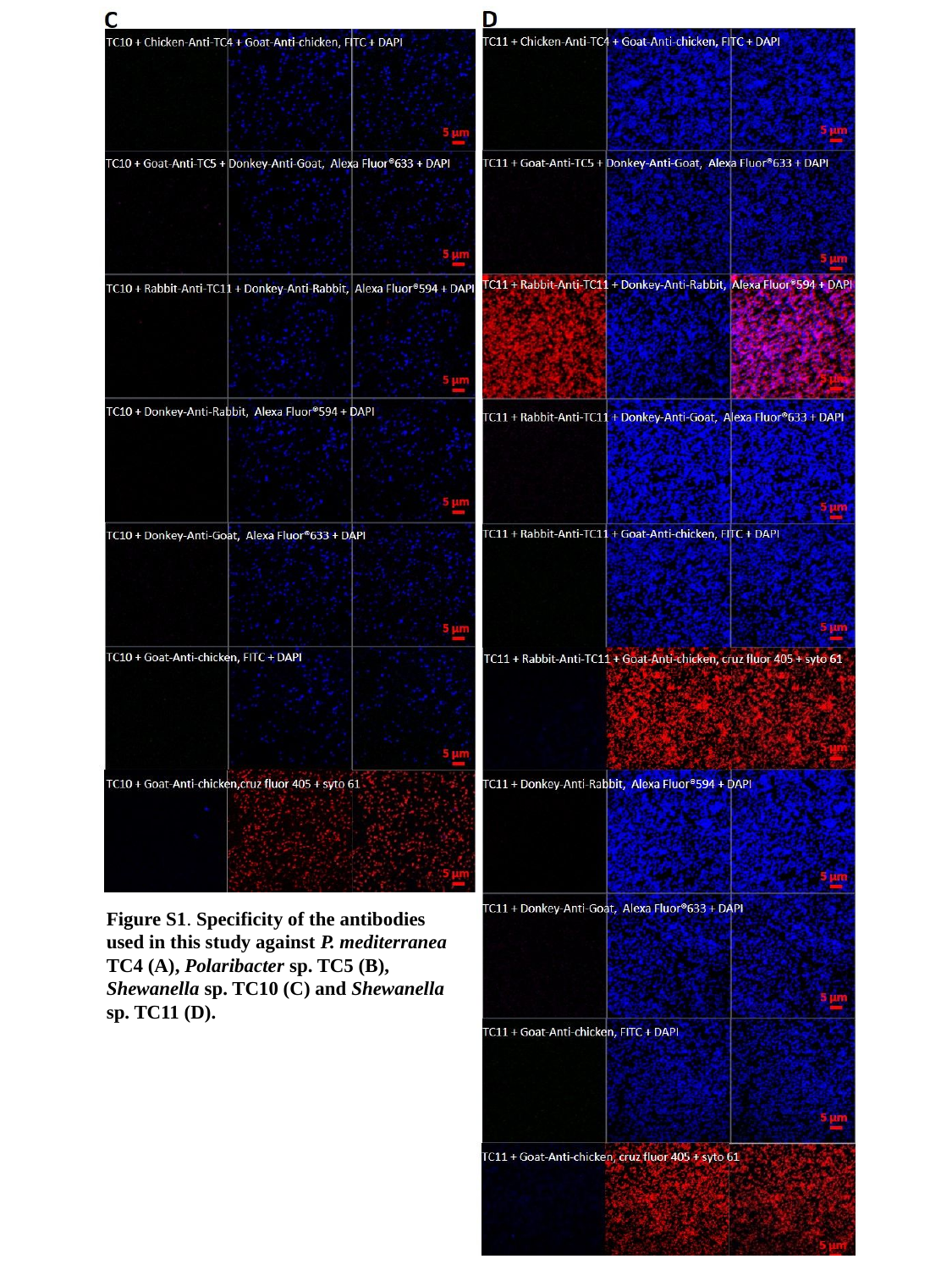

Figure S1. Specificity of the antibodies used in this study against P. mediterranea TC4 (A), Polaribacter sp. TC5 (B), Shewanella sp. TC10 (C) and Shewanella sp. TC11 (D).

## Slide 3
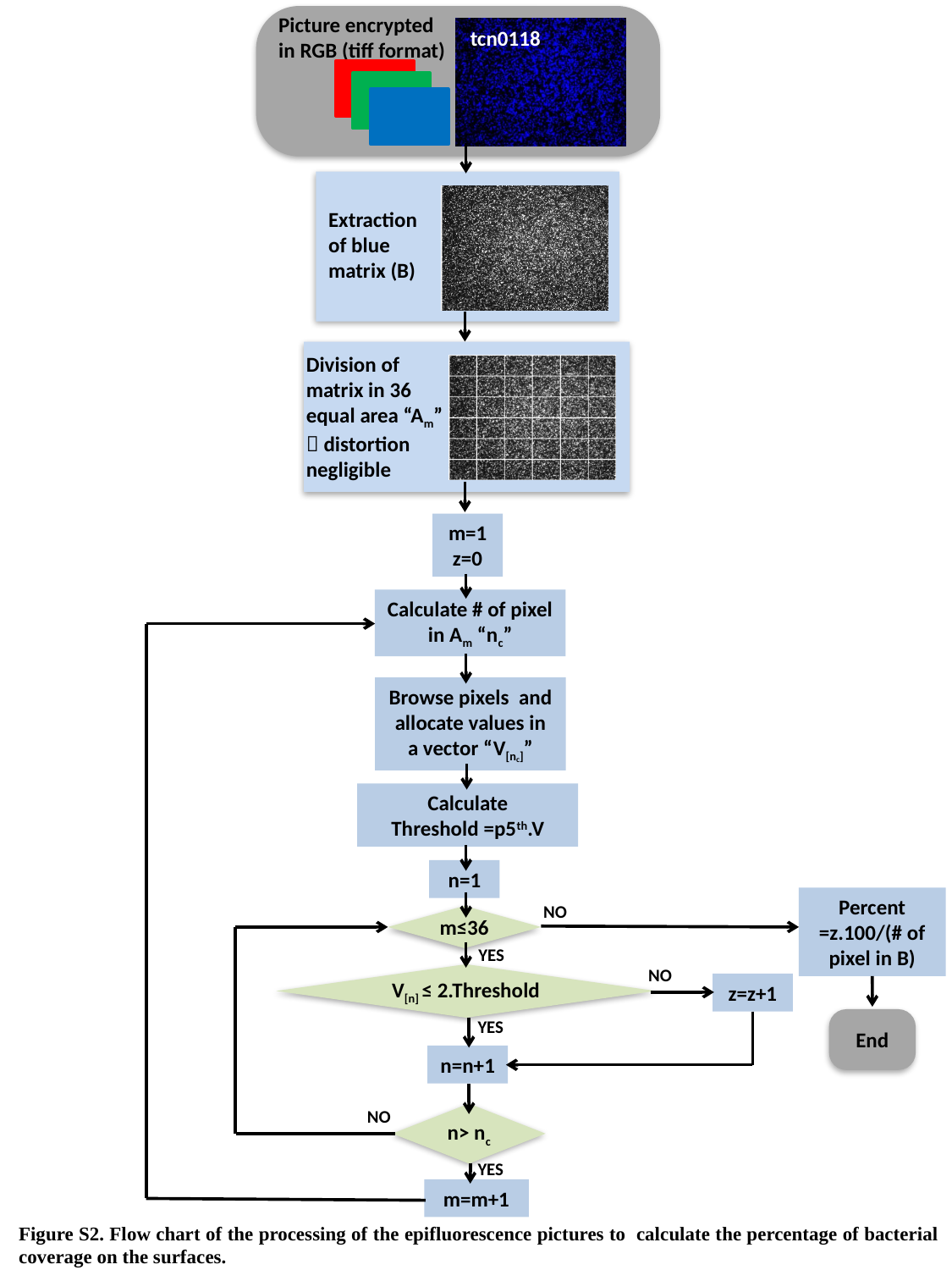

Picture encrypted in RGB (tiff format)
tcn0118
Extraction of blue matrix (B)
Division of matrix in 36 equal area “Am”
 distortion negligible
m=1
z=0
Calculate # of pixel in Am “nc”
Browse pixels and allocate values in a vector “V[nc]”
Calculate
Threshold =p5th.V
n=1
Percent =z.100/(# of pixel in B)
NO
m≤36
YES
NO
V[n] ≤ 2.Threshold
z=z+1
YES
End
n=n+1
NO
n> nc
YES
m=m+1
Figure S2. Flow chart of the processing of the epifluorescence pictures to calculate the percentage of bacterial coverage on the surfaces.

## Slide 4
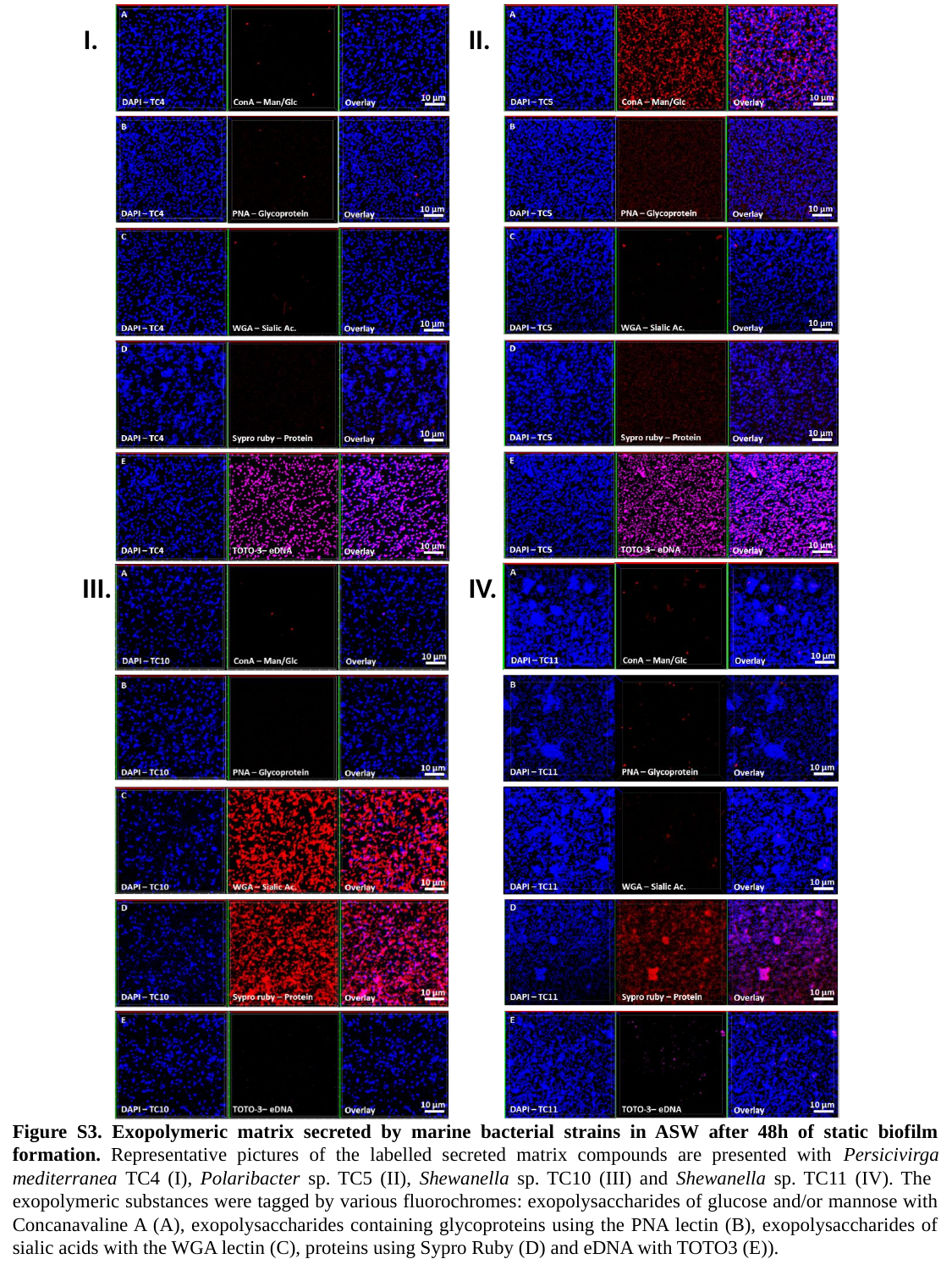

I.
II.
III.
IV.
Figure S3. Exopolymeric matrix secreted by marine bacterial strains in ASW after 48h of static biofilm formation. Representative pictures of the labelled secreted matrix compounds are presented with Persicivirga mediterranea TC4 (I), Polaribacter sp. TC5 (II), Shewanella sp. TC10 (III) and Shewanella sp. TC11 (IV). The exopolymeric substances were tagged by various fluorochromes: exopolysaccharides of glucose and/or mannose with Concanavaline A (A), exopolysaccharides containing glycoproteins using the PNA lectin (B), exopolysaccharides of sialic acids with the WGA lectin (C), proteins using Sypro Ruby (D) and eDNA with TOTO3 (E)).
